# A high neutrophil-to-lymphocyte ratio predicts hemorrhagic transformation of large atherosclerotic infarction in patients with acute ischemic stroke

**DOI:** 10.18632/aging.102752

**Published:** 2020-02-06

**Authors:** Wen-Bo Zhang, Ya-Ying Zeng, Fang Wang, Lin Cheng, Wen-Jie Tang, Xiao-Qiang Wang

**Affiliations:** 1 Department of Neurosurgery, Xinhua Hospital, Shanghai Jiaotong University School of Medicine, Shanghai, China; 2 Department of Neurology, The First Affiliated Hospital of Wenzhou Medical University, Wenzhou, China; 3 920th Hospital of PLA Joint Logistics Support Force, Kunming, China

**Keywords:** hemorrhagic transformation, the ratio of neutrophil to lymphocytes (N/L), large artery atherosclerosis, nomogram

## Abstract

Increasing evidence suggests that inflammation is associated with the development of acute ischemic stroke (AIS). The neutrophil-to-lymphocyte ratio (N/L) is an important marker of inflammation and is highly correlated with mortality in stroke patients in recent studies. The N/L of patients who experience hemorrhagic transformation (HT) after AIS is know, but any relationship between N/L and large artery atherosclerosis (LAA) remains unclear, this is our present topic. We enrolled 185 patients with LAA-type HT in the development cohort from a prospective, consecutive, hospital-based stroke registry to this end. We matched these patients to 213 LAA patients who did not develop HT as controls. The incidence of HT after LAA was significantly greater (P<0.01) in patients with higher N/L. We developed a predictive nomogram (incorporating age, systolic blood pressure, the National Institutes of Health Stroke Scale, and the N/L) for LAA patients. The predictive power was good (area under the curve, AUC: 0.832, 95%CI: 0.791–0.872). Our findings were further validated in a validation cohort of 202 patients with AIS attributable to LAA (AUC:0.836, 95%CI:0.781–0.891). In summary, a high N/L is associated with an increased risk for HT after LAA.

## INTRODUCTION

Acute ischemic stroke (AIS) is the third leading cause of disability and death after cardiovascular disease and cancer [1]. However, the current gold standard treatment for AIS is reperfusion therapy, which includes intravenous administration of recombinant tissue plasminogen activator (rt-PA) and endovascular treatment [2]. Hemorrhagic transformation (HT) is the most serious complication of AIS, caused by either the natural evolution of diease or reperfusion therapy of AIS [3]. Current treatment guidelines recommend anticoagulants, thrombolysis, and intravascular procedures for AIS patients. Despite these suggestions, these treatments have not been fully utilized due to concerns about HT, in particular in China [4]. Therefore, to determine whether thrombolytic therapy is safe, it is essential to understand why HT can develop after AIS.

Inflammatory after AIS is an important pathological process associated with post-cerebral ischemia [5]. Of the various white blood cells in peripheral circulation, neutrophils are important mediators of ischemic brain damage [6], and specific lymphocyte subtypes play key roles in inflammation and brain protection [7]. As the ratio of neutrophil to lymphocyte (N/L) fully reflects the balance between levels of these cells and thus their respective immune activities, the N/L better reflects the inflammation status of AIS patients than single neutrophil or lymphocyte counts. Recently, the N/L has been used to predict mortality after ischemic stroke and hemorrhagic stroke, and to predict transient ischemic attack [8]. The N/L is an important predictor of HT arising after AIS. One meta analysis found that a high N/L predicts the development of HT and 3-month mortality in patients with AIS [9], and was a significant risk factor for HT in patients receiving thrombolytic [10]. Large artery atherosclerosis (LAA) is the most common type of AIS, but it remains unclear whether the N/L predicts HT in patients with LAA. We explored this topic.

## RESULTS

### Patient characteristics

The general characteristics of the development and validation cohorts are detailed in Table 1. Of the 398 patients in the development cohort, 185 developed HT, and we matched them to 213 LAA patients without HT. Members of the HT group had an average age of 67.9 years, an average systolic blood pressure (SBP) of 150.4mmHg, an average of National Institute of Health Stroke Scale (NIHSS) of 6.6, and an average N/L of 5.9; In the non-HT group, the average age was 63.7 years, the average SBP was 156.6mmHg, the average NIHSS was 2.4, and the average N/L was 2.7.

**Table 1:** The general characteristics of the development and validation cohorts.

**Development cohort**					**Validation cohort**			
**Group**	**no-HT**	**HT**	P-value*		**Group**	**no-HT**	**HT**	P-value*
**N**	213	185			**N**	104	98	
**Age**	63.75 ± 10.58	67.91 ± 12.46	<0.001		**Age**	63.32 ± 10.45	69.19 ± 11.21	<0.001
**BMI**	23.85 ± 3.35	23.11 ± 3.24	0.051		**BMI**	23.90 ± 3.11	22.88 ± 3.53	0.045
**SBP**	156.59 ± 21.13	150.38 ± 22.35	0.011		**SBP**	153.71 ± 21.85	151.53 ± 23.18	0.669
**DBP**	83.68 ± 13.37	82.16 ± 13.66	0.214		**DBP**	82.89 ± 14.00	83.58 ± 15.08	0.84
**TG**	1.80 ± 1.05	1.69 ± 0.95	0.085		**TG**	1.77 ± 1.07	1.59 ± 0.76	0.161
**HDL**	1.14 ± 0.31	1.16 ± 0.33	0.49		**HDL**	1.14 ± 0.32	1.17 ± 0.26	0.428
**TC**	4.84 ± 1.17	4.79 ± 1.18	0.555		**TC**	4.80 ± 1.21	4.81 ± 1.13	0.826
**LDL**	2.92 ± 1.05	2.78 ± 0.91	0.306		**LDL**	2.82 ± 1.02	2.83 ± 0.87	0.735
**HCY**	8.61 ± 5.19	8.78 ± 5.65	0.722		**HCY**	8.71 ± 5.99	8.93 ± 5.67	0.393
**Neutrophil**	0.61 ± 0.09	0.70 ± 0.11	<0.001		**Neutrophil**	0.62 ± 0.10	0.71 ± 0.12	<0.001
**Lymphocyte**	0.28 ± 0.09	0.20 ± 0.12	<0.001		**Lymphocyte**	0.28 ± 0.10	0.19 ± 0.14	<0.001
**N/L**	2.71 ± 2.55	5.89 ± 6.89	<0.001		**N/L**	2.72 ± 1.86	6.03 ± 5.24	<0.001
**N+L**	0.89 ± 0.07	0.90 ± 0.09	0.017		**N+L**	0.89 ± 0.07	0.90 ± 0.11	0.423
**AST**	23.82 ± 12.61	31.11 ± 17.10	<0.001		**AST**	23.70 ± 13.98	30.26 ± 12.41	<0.001
**BUN**	5.41 ± 1.92	5.48 ± 1.79	0.728		**BUN**	5.25 ± 2.19	5.48 ± 1.84	0.264
**CR**	74.09 ± 19.52	86.86 ± 44.16	0.438		**CR**	75.23 ± 19.19	84.84 ± 40.72	0.864
**GH**	6.28 ± 1.45	6.05 ± 2.33	0.043		**GH**	6.21 ± 1.52	5.87 ± 1.95	0.175
**TB**	12.66 ± 7.96	14.51 ± 6.84	<0.001		**TB**	12.29 ± 6.39	15.53 ± 7.05	<0.001
**Albumin**	35.10 ± 1.98	37.91 ± 6.45	0.269		**Albumin**	35.10 ± 1.98	38.06 ± 6.03	0.286
**ALT**	23.79 ± 19.14	27.32 ± 24.10	0.318		**ALT**	23.85 ± 20.95	26.85 ± 22.56	0.712
**Platelet**	209.76 ± 62.58	206.34 ± 69.92	0.184		**Platelet**	208.41 ± 59.88	201.61 ± 68.76	0.151
**NIHSS**	2.38 ± 2.59	6.55 ± 4.99	<0.001		**NIHSS**	2.45 ± 2.79	6.60 ± 4.88	<0.001
Sex			0.907		Sex			0.838
male	152 (71.36%)	133 (71.89%)			male	74 (71.15%)	71 (72.45%)	
female	61 (28.64%)	52 (28.11%)			female	30 (28.85%)	27 (27.55%)	
**Smoke**			0.086		**Smoke**			0.379
smoking now	99 (46.70%)	72 (39.78%)			smoking now	48 (46.15%)	35 (36.46%)	
quit smoking	33 (15.57%)	21 (11.60%)			quit smoking	17 (16.35%)	19 (19.79%)	
no smoking	80 (37.74%)	88 (48.62%)			no smoking	39 (37.50%)	42 (43.75%)	
**Drink**			<0.001		**Drink**			<0.001
drinking now	170 (84.58%)	66 (36.46%)			drinking now	84 (84.00%)	39 (40.62%)	
quit drinking	16 (7.96%)	13 (7.18%)			quit drinking	8 (8.00%)	10 (10.42%)	
no drinking	15 (7.46%)	102 (56.35%)			no drinking	8 (8.00%)	47 (48.96%)	
**Stroke history**			0.013		**Stroke history**			0.02
no	193 (91.90%)	155 (83.78%)			no	97 (93.27%)	81 (82.65%)	
yes	17 (8.10%)	30 (16.22%)			yes	7 (6.73%)	17 (17.35%)	
**HP**			0.459		**HP**			0.728
no	63 (29.86%)	61 (33.33%)			no	36 (34.62%)	31 (32.29%)	
yes	148 (70.14%)	122 (66.67%)			yes	68 (65.38%)	65 (67.71%)	
**DM**			0.696		**DM**			0.67
no	153 (72.86%)	138 (74.59%)			no	78 (75.00%)	76 (77.55%)	
yes	57 (27.14%)	47 (25.41%)			yes	26 (25.00%)	22 (22.45%)	
**AF**			0.211		**AF**			0.241
no	206 (97.17%)	175 (94.59%)			no	100 (96.15%)	90 (91.84%)	
yes	6 (2.83%)	10 (5.41%)			yes	4 (3.85%)	8 (8.16%)	

Note: In the Development cohort, univariate analysis found that Age, Neutrophil, Lymphocyte, N/L, N+L, AST, TB, GH and NHISS in patients with HT had statistically significant differences in patients who did not develop HT.

Abbreviation: BMI: Body Mass Index; SBP: Systolic blood pressure; DBP: Diastolic blood pressure; TG: Triglyceride; TC: Total cholesterol; HDL: High density lipoprotein; LDL: Low density lipoprotein; HCY: Homocysteine; N/L: Neutrophil/ Lymphocyte; N+L: Neutrophil + Lymphocyte; AST: Aspartate aminotransferase; ALT: Alanine aminotransferase; CR: Creatinine; BUN: Blood urea nitrogen; GH: Glycated hemoglobin; TB: Total bilirubin; HP: Hypertension; DM: Diabetes mellitus; AF: Atrial fibrillation.

Univariate and multivariate were used to identify factors potentially prognostic factors of HT, we used logistic regression to this end. The two groups differed significantly in terms of age, SBP, and NIHSS (Table 2). On this basis, we have constructed three models, Model 1 contained these parameters, and models 2 and 3 contained neutrophil and lymphocyte counts and the N/L, respectively. Through comparison, we will select the best model to build nomogram.

**Table 2:** Univariate analyses for the potential factors associated with hemorrhagic transformation by Logistic regression.

	**Model1**				**Model2**				**Model3**		
	**P**	**OR**	**95% CI**		**P**	**OR**	**95% CI**		**P**	**OR**	**95% CI**
**Age**	<0.01	1.04	1.018-1.066	**Age**	<0.01	1.04	1.02-1.07	**Age**	<0.01	1.04	1.02-1.07
**SBP**	<0.01	0.97	0.961-0.986	**SBP**	<0.01	0.97	0.96-0.99	**SBP**	<0.01	0.97	0.96-0.98
**TB**	0.05	1.03	0.999-1.069	**TB**	0.06	1.03	1.00-1.07	**TB**	0.28	1.02	0.98-1.06
**AST**	0.49	1.02	1.000-1.041	**AST**	0.05	1.02	1.00-1.04	**AST**	0.12	1.02	1.00-1.04
**GH**	0.54	0.95	0.808-1.119	**GH**	0.55	0.95	0.81-1.12	**GH**	0.81	0.98	0.82-1.16
**NIHSS**	<0.01	11.92	5.198-27.356	**NIHSS**	<0.01	11.82	5.14-27.22	**NIHSS**	<0.01	1.32	1.22-1.44
				**N+L**	0.81	1.73	0.02-157.94	**N/L**	0.03	1.12	1.01-1.25

Note: Logistic regression analysis found that patients’ Age, SBP, and HIHSS are independent risk factors for HT. On this basis, we have established three models, and Model 2 and Model 3 add two variables, N+L and N/L, respectively.

Abbreviation: SBP: Systolic blood pressure; N/L: Neutrophil/ Lymphocyte; N+L: Neutrophil + Lymphocyte; AST: Aspartate aminotransferase; GH: Glycated hemoglobin; TB: Total bilirubin.

**Figure 1:**
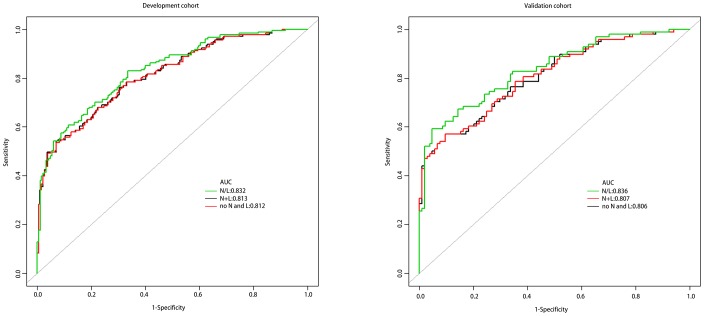
**The ROC curves of the three models.**

**Figure 2:**
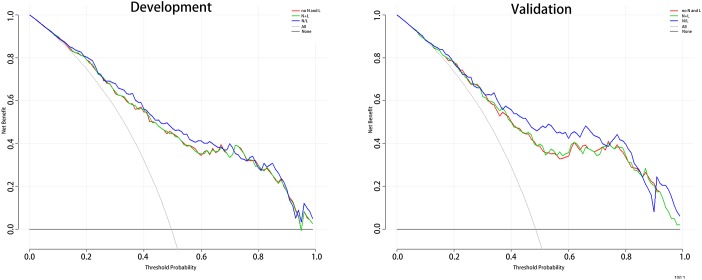
**The DCA for 3 models to predict the correct diagnosis of HT patients.** Abbreviation: DCA: Decision Curve Analysis; HT: hemorrhagic transformation. Note: The net benefit value of model3 is higher than the other two groups.

In order to explore the best connection between neutrophil, lymphocyte and HT, we have produced three prediction models for this purpose. Model 1 includes age, SBP, NIHSS; model 2 includes age, SBP, NIHSS, neutrophil+lymphocyte (N+L); and model 3 includes age, SBP, NIHSS, N/L. Receiver operating characteristic (ROC) curves are shown in Figure 1, and the three AUCs were 0.812, 0.813 and 0.832, respectively. We performed Decision Curve Analyses (DCAs) for the three models. Figure 2 shows that all three DCA curves correctly diagnosed HT, but the net benefit afforded by model 3 was the highest.

**Figure 3:**
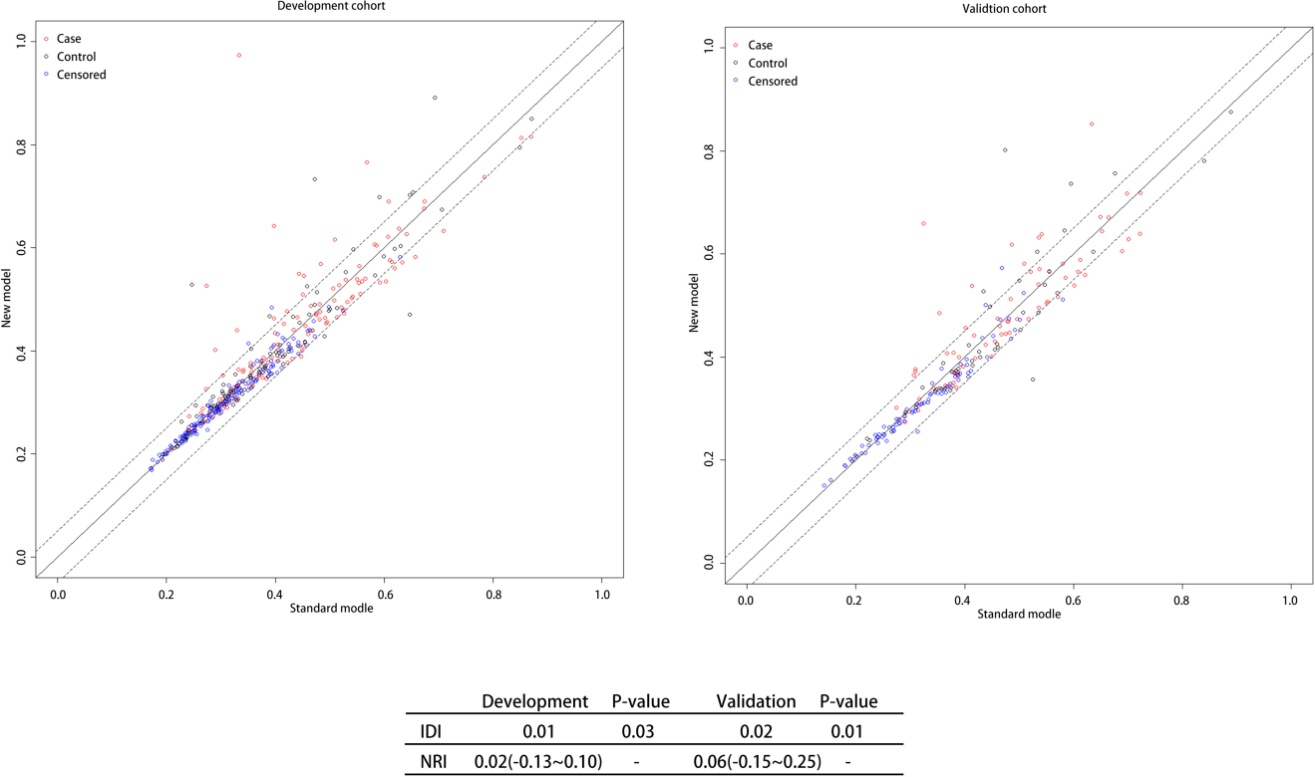
**NRI and IDI between N/L and N+L groups.** Note: Both NRI and IDI show that Model3 has a higher predictive power than Model2. Abbreviation: NRI: Net reclassification index; IDI: Integrated Discrimination Improvement; N/L: Neutrophil/ Lymphocyte; N+L: Neutrophil + Lymphocyte.

**Figure 4:**
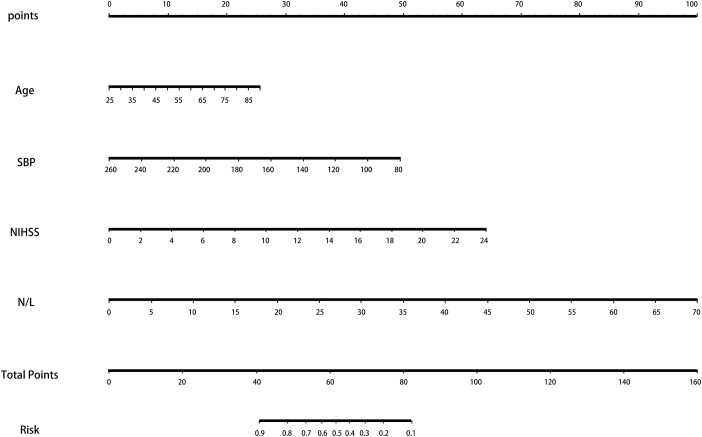
**The nomogram for patients with LAA-type HT.** Note: To use the nomogram, an individual patient’s value is located on each variable axis, and a line is drawn upward to determine the number of points received for each variable value. The sum of these numbers is located on the Total Points axis, and a line is drawn downward to the survival axes to determine the likelihood of HT. Abbreviation: LAA: large artery atherosclerosis; HT: hemorrhagic transformation.

The ROC and DCA curves showed that the predictive accuracies of model 2 and model 3 were higher than that of model 1. In order to compare the differences in predictive performance between model 2 and model 3, we calculated Net Reclassification Indexs (NRIs) and Integrated Discrimination Improvements (IDIs), as shown in Figure 3. The IDI approach showed that the predictive ability of model 3 was significantly better than that of model 2 for both the development cohort (IDI = 0.01, P = 0.03) and the validation cohort (IDI = 0.02, P = 0.01). The NRI showed that the predictive capacities of the two models for both groups were good, but model 3 was better. The NRIs for the development and validation cohorts were 0.02(-0.13–0.10) and 0.06(-0.15–0.25), respectively (Figure 3). Thus, we selected model 3 to establish a nomogram for patients with LAA-type HT (Figure 4). Multivariate logistic regression indicated that age, SBP, NIHSS and N/L were all useful predictors. The model evidenced good predictive power (AUC:0.832, 95%CI:0.791–0.872). In the validation cohort, the model also predicted HT well (AUC:0.836, 95%CI:0.781–0.891).

According to the recommendations of the European Cooperative Acute Stroke Study (ECASS) [11], HT is divided into HI and PH, and the patient's baseline data are shown in Table 3. The HI group had an average age of 68.9 years, an average SBP of 152.7mmHg, an average NIHSS of 7.63, and an average N/L of 5.42. The PH group had an average age of 66.3 years, an average SBP of 146.7mmHg, an average of NIHSS of 5.9, and an average N/L of 6.67.

**Table 3:** The patient's baseline data of PH and HI.

**HT**	**HI**	**PH**	**P-value**
N	114	71	
**Age**	68.90 ± 12.11	66.31 ± 12.94	0.249
**BMI**	23.15 ± 2.71	23.00 ± 4.23	-
**SBP**	152.66 ± 21.19	146.73 ± 23.79	0.063
**TC**	4.75 ± 1.13	4.87 ± 1.26	-
**TG**	1.60 ± 0.81	1.85 ± 1.13	-
**HDL**	1.14 ± 0.29	1.19 ± 0.38	-
**DBP**	82.72 ± 13.59	81.27 ± 13.80	-
**LDL**	2.81 ± 0.98	2.73 ± 0.78	-
**HCY**	9.61 ± 5.95	7.27 ± 4.78	-
**Neutrophil**	0.69 ± 0.11	0.72 ± 0.11	0.065
**Lymphocyte**	0.20 ± 0.10	0.19 ± 0.15	0.063
**N/L**	5.42 ± 7.20	6.67 ± 6.31	0.076
**N+L**	0.89 ± 0.05	0.91 ± 0.12	0.784
**TB**	13.91 ± 6.65	15.48 ± 7.08	-
**Albumin**	36.94 ± 4.42	39.51 ± 8.65	-
**ALT**	26.81 ± 23.38	28.14 ± 25.38	-
**AST**	30.93 ± 17.25	31.41 ± 16.98	-
**BUN**	5.59 ± 1.88	5.29 ± 1.60	-
**CR**	84.49 ± 42.64	90.81 ± 46.66	-
**GH**	6.00 ± 1.82	6.13 ± 3.03	-
**Platelet**	214.59 ± 73.57	192.90 ± 61.71	-
**NIHSS**	5.90 ± 5.03	7.63 ± 4.78	0.01
**Sex**			-
male	79 (69.30%)	54 (76.06%)	
female	35 (30.70%)	17 (23.94%)	
**Smoke**			-
smoking now	43 (38.39%)	29 (42.03%)	
quit smoking	13 (11.61%)	8 (11.59%)	
no smoking	56 (50.00%)	32 (46.38%)	
**Drink**			-
drinking now	38 (33.93%)	28 (40.58%)	
quit drinking	9 (8.04%)	4 (5.80%)	
no drinking	65 (58.04%)	37 (53.62%)	
**Stroke history**			-
no	94 (82.46%)	61 (85.92%)	
yes	20 (17.54%)	10 (14.08%)	
**HP**			-
no	33 (29.46%)	28 (39.44%)	
yes	79 (70.54%)	43 (60.56%)	
**DM**			-
no	83 (72.81%)	55 (77.46%)	
yes	31 (27.19%)	16 (22.54%)	
**AF**			-
no	107 (93.86%)	68 (95.77%)	
yes	7 (6.14%)	3 (4.23%)	

Abbreviation: BMI: Body Mass Index; SBP: Systolic blood pressure; DBP: Diastolic blood pressure; TG: Triglyceride; TC: Total cholesterol; HDL: High density lipoprotein; LDL: Low density lipoprotein; HCY: Homocysteine; N/L: Neutrophil/ Lymphocyte; N+L: Neutrophil + Lymphocyte; AST: Aspartate aminotransferase; ALT: Alanine aminotransferase; CR: Creatinine; BUN: Blood urea nitrogen; GH: Glycated hemoglobin; TB: Total bilirubin; HP: Hypertension; DM: Diabetes mellitus; AF: Atrial fibrillation.

**Figure 5:**
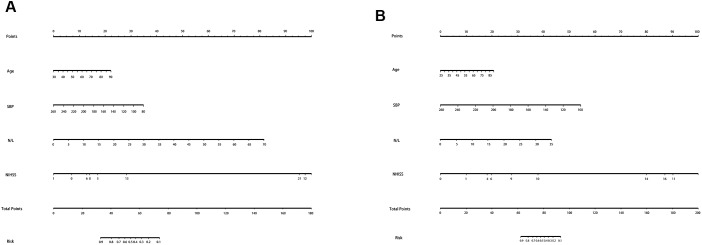
**The nomogram for HI and PH in LAA patients.** Abbreviation: LAA: large artery atherosclerosis.

**Figure 6:**
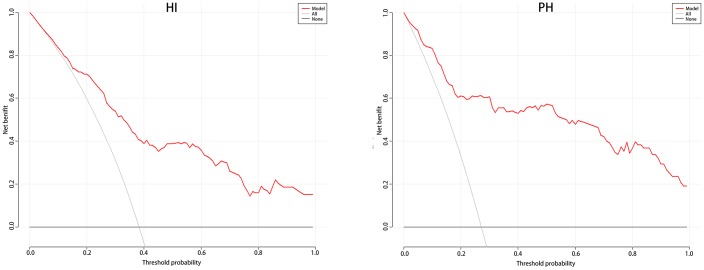
**The DCA for HI and PH in LAA patients.** Abbreviation: DCA: Decision Curve Analysis; LAA: large artery atherosclerosis.

Table 3 shows no differences in age, SBP, NIHSS, and N/L between the two groups. Therefore, we used these four predictors to create a nomogram predicting the probability of HI and PH in LAA patients (Figure 5). For the HI group, the AUC was 0.821(95%CI: 0.773–0.867); for the PH group, the AUC was 0.896(95%CI: 0.844–0.931). In the validation cohort, their AUCs are 0.794 and 0.882, respectively, which also indicated good model repeatability and very good predictive power in both populations. DCA in both groups revealed a good net benefit (Figure 6).

## DISCUSSION

Using a development cohort derive from the prospective continuous hospital stroke registry of the First Affiliated Hospital of Wenzhou Medical, we found that N/L is a good predictor of HT in patients with LAA. Our external validation cohort, from Xinhua Hospital (Shanghai, China) and 920^th^ Hospital (Kunming, China), confirmed that the model exhibited good verifiability. Relationships between neutrophil counts, on the one hand, and AIS, on the other, have been widely reported. The neutrophil count in patients with cerebral ischemia and a high N/L are independently associated with HT [12], because infection after stroke can cause problems via many different mechanisms. Neutrophils are an important source of matrix metalloprotein-9 (mmp-9), which acts on tight junction proteins, opens the blood-brain barrier (BBB) in the vascular lumen, and is absorbed by endothelial cells and then incorporated into basement membranes [2]. In a rat model of cerebral ischemia, preventing neutrophil infiltration reduced MMP-9 release in the brain. Neutrophil inhibition or destruction preserves the BBB and educes the incidence of HT in AIS patients [13, 14]. Conversely, when mouse neutrophil numbers increase in the presence of lipopolysaccharide or granulocyte colony-stimulating factor, the associated BBB destruction and elevated MMP-9 level increase the incidence of rt-PA-associated HT [15]. Therefore, neutrophil-derived MMP can trigger HT in AIS patients. Apart from MMP, other factors released after stroke, including reactive oxygen species(ROS), cathepsin G, proteases, myeloperoxidase, elastase, chemokines and cytokines, may also destroy neurovascular units, ultimately increasing the permeability of the BBB and the risk for HT [16]. BBB destruction is accompanied by vasogenic edema and hemorrhage [17, 18]. Soon after ischemia develops circulating neutrophils are recruited to the site of the brain injury, further increasing BBB destruction and damaging other tissue via various mechanisms [19, 20]. Therefore, increased numbers of neutrophils significantly increase the risk for HT

The lymphocyte counts an indicator of general health, is affected by acute physiological stress [21]. Relative reductions in lymphocyte reflect activation of the cortisol-induced stress response and sympathetic excitation, which increase the production of pro-inflammatory cytokines that aggravate ischemic injury [22]. In experiments, specific lymphocyte subtypes played key roles in eliminating inflammatory responses, serving as the principal immunomodulators protecting the brain in AIS patients [23].

The N/L reflects the balance between neutrophil and lymphocyte levels, and can fully reflect the immune level. In this sense, the N/L is better than the neutrophil count or lymphocyte count alone for predicting HT. However, the release of inflammatory cytokines from neutrophils can induce apoptosis of lymphocytes, which suggests that the N/L may not only reflect the number of neutrophils and lymphocytes, but also reflect the over-activation of neutrophils, which increases the disparity in number between these two types of white blood cells. This emphasizes the utility of the N/L [24]. We found that the model containing the N/L was more predictive than the other models. Earlier reports found that the risk for HT in high-N/L patients was 1.53-fold that in low-N/L patients, and the 3-month mortality rate was 1.1-fold greater. Lymphocytes accumulated in the ischemic brain 3-6 days after stroke, later than neutrophils. Neutrophil and lymphocyte counts yield different prognostic predictions in patients with acute cerebral infarction. Although higher neutrophil numbers may increase the severity of initial strokes, lower lymphocyte numbers are associated with poorer long-term outcomes [25]. Therefore, a high N/L in peripheral blood indicates poor prognosis in AIS patients.

LAA is a key subtype of the Trial of Org 10172 in Acute Stroke Treatment (TOAST) classification system, accounted for 50.74% of all AIS in the work of Deng [26]. It was also the most common AIS subtype in our research. We found that N/L predicted HT in LAA patients, and afforded superior predictive ability when N/L is included in the nomogram. HT can be divided into HI and PH. Although the two groups did not differ significantly in terms of the N/L, the PH group had a higher DCA and AUC, which indicates that the N/L may directly reflect HT severity.

In conclusion, this work suggests that a high level of N/L may be associated with an increased risk for HT in LAA patients. The positive correlation between the risk for HT and N/L should be considered when treating such patients.

## MATERIALS AND METHODS

This study was approved by the Ethics Committee of Xinhua Hospital and the Ethics Committee of the First Affiliated Hospital of Wenzhou Medical University, and the Ethics Committee of 920th Hospital of PLA Joint Logistics Support Force and conformed to the Helsinki Declaration. From 2012 to 2018, there were 287 AIS patients with HT were admitted to the First Affiliated Hospital of Wenzhou Medical University, 185 of whom had LAA. They were matched to 213 LAA patients without HT; these 398 patients served as the development cohort. A total of 202 LAA patients were included in the external validation cohort.

### Exclusion criteria

1, excluding other vascular infarction, cerebral venous thrombosis; 2, patients with transient ischemic attack, cerebral hemorrhage or subarachnoid hemorrhage; 3, history of oral anticoagulants or antiplatelet drugs before admission; 4, patients undergoing thrombolysis or thrombectomy after admission; 5, Any factors that affect inflammation indicators, including severe infection or antibiotic use before admission, blood disease, immunosuppression use, glucocorticoid use, or severe liver and/or kidney disease, as well as recent trauma and/or major surgery, are excluded. All patients were admitted within 7 days of stroke onset, and their demographic, chronic disease, hematologic parameters, and imaging findings were collected using standardized data records.

### Clinical and laboratory assessments

All blood indicators were assessed on the morning of the second day of admission after overnight fasting. Doppler ultrasonography was performed within 48 hours of admission. CT examinations were completed within 24 hours of admission; Repeat CT and MRI were performed prior to discharge. Cerebrovascular events were classified according to the TOAST criteria (Table 4) [27].

**Table 4:** Features of TOAST Classification of Subtypes of Ischemic stroke.

Features	**Subtype**			
	**Large-artery atherosclerosis**	**Cardioembolism**	**Small-artery occlusion**	**other cause**
**Clinical**				
Cortical or cerebeller dysfunction	+	+	-	+/-
Lacunar sydrome	-	-	+	+/-
**Imaging**				
Cortical, cerebeller, brain-stem, or subcortical infarct>1.5cm	+	+	-	+/-
Brain-stem, or subcortical infarct<1.5cm	-	-	+/-	+/-
**Tests**				
Stenosis of extracranial internal carotid artery	+	-	-	-
Cardiac source of embolism	-	+	-	-
Other abnormality on tests	-	-	-	+

### Classification criteria

The diagnostic criteria for LAA were those of TOAST [28]. HT typing followed the recommendation of the ECASS [11]; HT was divided into HI and PH. HI was defined as small petechiae along the margins of the infarct (HI1) or as more confluent petechiae within the infarcted area but without any space-occupying effect (HI2). Parenchymal hematoma was defined as hematoma in ≤30% of the infarcted area with some slight space-occupying effect (PH1) or as dense hematoma in ≥30% of the infarcted area with substantial space-occupying effect or any hemorrhagic lesion outside the infarcted area (PH2).

### Follow-up

All patients underwent CT every 3 days after admission, 2 weeks after discharge, and 1 month after discharge. Two independent investigators evaluated all clinical data blind; any disagreement was resolved by a third researcher.

### Statistical analysis

Patient baseline data and risk factors were statistically analyzed by SPSS. Categorical variables were compared with the chi-square test, and continuous variable were compared with the Kruskal Wallis rank sum test. If the count variable has a theoretical number <10, comparisons were made with Fisher's exact probability test. Logistics regression analysis was used for multivariate analyses. The nomogram was constructed via such analyses performed with rms26 in R version. After logistic regression analysis and calculation of risk factors, we ranked nomogram variables using their P values and effect values and assessed the performance of the nomogram by calculating the AUC. The larger the AUC, the more accurate the prognosis is. We also compared the predictive powers of different models by calculating IDI, NRI, and DCA values. P<0.05 was considered statistically significant and all calculations were based on SPSS version 22.0 software and R.
